# Immune Dysregulation, Compartment-Specific Biology, and Therapeutic Biomarkers in Mycosis Fungoides and Sézary Syndrome

**DOI:** 10.3390/cells15171576

**Published:** 2026-08-30

**Authors:** Tiantian Zhang, Weili Xue, Simo Du, Jiahe Zhao, Yumeng Zhang

**Affiliations:** 1 Department of Internal Medicine, University of Central Florida/HCA, Pensacola, FL 32514, USA; 2 Department of Oncology, The First Affiliated Hospital of Zhengzhou University, Zhengzhou 450000, China; 3 Jacobi Medical Center, Albert Einstein College of Medicine, Bronx, NY 10461, USA; 4 Flushing Hospital Medical Center, Flushing, NY 11355, USA; 5 Department of Malignant Hematology, Moffitt Cancer Center, Tampa, FL 33612, USA

**Keywords:** cutaneous T-cell lymphoma, mycosis fungoides, Sézary syndrome, immune dysregulation, tumor microenvironment, immunotherapy, biomarkers

## Abstract

Cutaneous T-cell lymphoma (CTCL), including mycosis fungoides (MF) and Sézary syndrome (SS), is defined by malignant CD4+ T-cell clones that evolve within and remodel the skin and systemic immune compartments. For disease biology and immune remodeling, we prioritize evidence from human primary samples; for therapeutic implications, we integrate clinical-trial and translational biomarker studies. We organize evidence by compartment (skin vs. blood) and, within skin, by stage (patch/plaque vs. tumor). Early patch/plaque MF often shows features consistent with constrained inflammation: skin-resident memory T-cell (TRM)-like malignant clones are in a Th1-leaning microenvironment with relatively preserved cytotoxic surveillance. Progression to tumor-stage MF (and/or large-cell transformation) is frequently associated with clonal dominance, Th2 cytokine polarization, upregulation of checkpoint pathways, and remodeling of myeloid/fibroblast populations. SS is typically associated with leukemic clonal dominance, systemic Th2 skewing, and broad impairment of cytotoxic immune control. These trajectories support an interpretive framework that aligns immune-directed therapies with compartment- and stage-associated biology; these include strategies that (i) deplete malignant clones (e.g., CCR4, CD30, KIR3DL2 targeting), (ii) re-engage exhausted effectors (PD-1/PD-L1 axis), (iii) reprogram cytokine balance (IFN-γ, IL-12, extracorporeal photopheresis), or (iv) suppress malignant signaling programs with secondary immune effects (JAK/STAT inhibition). We highlight potential candidate predictive biomarkers at varying levels of maturity, most of which require prospective validation, including antigen density, compartmental tumor burden, tumor cell fraction, effector-cell substrate, and interferon/cytokine signatures.

## 1. Introduction

Cutaneous T-cell lymphoma (CTCL) encompasses a group of lymphoproliferative disorders characterized by the accumulation of malignant T cells in the skin. CTCL accounts for approximately 70% of primary cutaneous lymphomas, with mycosis fungoides (MF) representing the major subtype (>60%), and Sézary syndrome (SS) comprising a smaller but clinically aggressive subset (approximately 5%) [1,2,3]. In MF, malignant T cells exhibit epidermotropism, producing patches and plaques that can mimic inflammatory dermatoses; early-stage MF often follows an indolent course with long median survival. A subset of patients progresses to tumor-stage disease and may develop large-cell transformation (LCT), a transition associated with nodal/visceral dissemination and poor outcomes [4,5,6,7]. SS arises de novo or evolves from MF and is characterized by erythroderma, circulating malignant T cells, and lymphadenopathy, with a median survival of 2–4 years [4,5].

Immune dysregulation is central to MF/SS pathogenesis and progression. Early-stage patch/plaque MF is often associated with relatively preserved Th1-associated immune features and cytotoxic surveillance, whereas advanced tumor-stage or transformed MF and SS show progressive Th2 polarization, impaired cytotoxic function, and increased immune checkpoint signaling [8,9,10,11,12,13,14,15,16,17,18,19]. This review focuses specifically on MF and SS, recognizing them as biologically and clinically distinct entities within the broader CTCL spectrum. Whether MF and SS are biologically distinct entities or represent overlapping points on a continuum remains debated, with cell-of-origin and genomic data involved on both sides [20]. For disease biology and immune remodeling, we prioritize evidence from human primary samples, including tissue- and blood-based immunophenotyping, flow cytometry, cytokine profiling, T-cell receptor (TCR) clonality testing by polymerase chain reaction (PCR) and next-generation sequencing (NGS), whole-exome/whole-genome sequencing (WES/WGS), and bulk and single-cell transcriptomic analyses. For therapeutic implications, we integrate clinical-trial and potential translational biomarker evidence. We organize data by compartment (skin vs. blood) and by skin stage (patch/plaque vs. tumor), then interpret immune-based therapies in the context of these stage- and compartment-dependent immune changes.

Because the MF/SS literature spans randomized trials, prospective early-phase studies, retrospective cohorts, translational immune profiling, and mechanistic hypotheses, we organize therapeutic and biomarker evidence according to maturity. Throughout, we distinguish approved or established therapies supported by phase III or mature prospective data; therapies supported by phase I/II studies; retrospective or translational biomarker associations; and hypothesis-generating concepts that require prospective validation. We apply the same graded framework to candidate biomarkers, separating those used clinically for treatment selection or disease assessment from those that remain exploratory or mechanistically motivated.

### Review Methodology

This article is a narrative review informed by a structured, non-systematic literature search. PubMed/MEDLINE and ClinicalTrials.gov were searched from database inception through 20 August 2026, supplemented by backward reference searching of key reviews, pivotal trials, and regulatory or sponsor reports when peer-reviewed full reports were not yet available. Search concepts combined controlled vocabulary and free-text terms for “cutaneous T-cell lymphoma,” “mycosis fungoides,” or “Sézary syndrome” with terms related to immune dysregulation, tumor microenvironment, skin, blood, stage, transformation, cytokines, T-cell receptor clonality, genomics, transcriptomics, single-cell analysis, biomarker, immunotherapy, and the individual therapeutic targets discussed in this review. Searches were iteratively updated during manuscript revision.

For disease biology, priority was given to English-language studies of human primary skin or blood samples from patients with MF or SS, particularly studies that specified disease subtype, stage, compartment, sampling structure, and assay. Mixed-CTCL cohorts were included only when MF/SS-specific findings could be extracted or when the mixed composition was explicitly acknowledged. For therapeutic sections, prospective clinical trials and mature regulatory data were prioritized, followed by retrospective cohorts and translational biomarker analyses; preclinical studies were used only to support mechanistic context. Conference abstracts and sponsor communications were retained only for recent investigational agents lacking a complete peer-reviewed report and are identified as lower-maturity evidence. Studies focused exclusively on other primary cutaneous lymphomas, non-human models without direct human corroboration, isolated case reports without mechanistic relevance, and non-English reports lacking sufficient extractable data were excluded. This was not a systematic review: study selection was not performed in duplicate; a PRISMA flow diagram and formal risk-of-bias assessment were not used, and no meta-analysis was undertaken.

Human-sample evidence in Table 1 was graded based on the biological observation rather than on assay maturity. “Strong” indicates a concordant finding reproduced in multiple independent human cohorts, ideally across complementary assays or with functional support; “Moderate–strong” indicates a reproducible principal finding for which one or more components rely on fewer cohorts or less complete compartment matching; “Moderate” indicates generally concordant evidence from a limited number of cohorts or modest sample sizes; and “Limited” indicates a single or exploratory cohort, substantial cohort or assay heterogeneity, or discordant findings. Methodological maturity was not used by itself to increase the grade. Therapeutic evidence was classified separately according to development maturity, as defined in Table 2. Study designs and citation assignments were checked against the primary reports where available.

## 2. Skin Compartment

### 2.1. Patch/Plaque MF (Early-Stage): Constrained Inflammation Around TRM-like Malignant Clones

Early-stage MF is characterized by a relatively low malignant burden within a reactive immune microenvironment. Multiple human-sample studies support a skin-resident memory T-cell (TRM)-like phenotype of malignant cells in MF, with expression of skin-homing and tissue-retention programs (e.g., CCR4 and CLA, and TRM-associated markers including CD69 and CD103) and limited expression of recirculation markers (CCR7 and L-selectin), consistent with clinical confinement to the skin in many early cases [20,21,22]. This compartmentalization is reinforced by keratinocyte and stromal chemokine programs that support epidermotropism.

Early MF lesions can appear “inflammatory” because malignant and reactive immune cells are intermingled, and antigen-presenting cell-driven cues may contribute to chronic activation states even when the initiating antigen(s) are undefined [21,23]. Malignant MF cells may show an aberrant immunophenotype (including variable loss of pan-T-cell markers such as CD2/CD5/CD7) and can express immune-regulatory or checkpoint-associated markers (e.g., PD-1 and FOXP3) in subsets, which becomes increasingly relevant as immune escape emerges [22,24].

Immunologically, early patch/plaque MF tends to retain a Th1-leaning microenvironment. Human-sample analyses show higher IFN-γ-associated signals in early lesions, with IL-12 signaling supporting cytotoxic CD8+ T-cell and NK-cell activity [12,24,25]. In several cohorts, CD8+ tumor-infiltrating lymphocytes (TILs) are more abundant in early disease and associate with improved outcomes, and their density declines with progression [14]. Clinical trials of IL-12 treatment in CTCL patients have shown 50% response rates, and responding lesions demonstrated increased CD8+ and TIA-1+ infiltrates, directly linking a Th1-restoring intervention to augmented cytotoxic substrate in tissue [26]. Sarris et al. also showed that IFN-γ secreted by lymphoid cells induces keratinocytes to produce IP-10, attracting CD4+ T cells to the epidermis and providing a mechanistic explanation for epidermotropism in early lesions [12]. Similarly, others noted a stage-related decline in IFN-γ and reduced epidermotropism with progression [12,13].

Regulatory circuits in early MF may reflect control of inflammation rather than uniform immunosuppression. In human tissue studies, FOXP3+ regulatory T cells (Tregs) have been variably reported, with multiple reports associating higher FOXP3+ frequencies in early lesions with favorable outcomes, consistent with restraint of pathologic inflammation in this context [27,28,29]. Regulatory B cells (Bregs), including IL-10-producing subsets, are also reported in CTCL and may decline with progression, suggesting that early MF can represent an immune state of constrained, locally contained inflammation rather than global immune collapse [30].

### 2.2. Tumor-Stage MF (±Large-Cell Transformation): Immune Escape, Th2 Polarization, and Stromal/Myeloid Remodeling

As MF progresses to tumor-stage disease, malignant clones expand and acquire additional genetic lesions that reinforce survival, proliferation, and immune evasion. Across genomic studies using human samples, recurrent alterations involve TCR signaling (e.g., PLCG1, PRKCQ, CD28), JAK/STAT pathway genes (STAT3, STAT5B, JAK1, JAK3), NF-κB signaling, and chromatin modifiers (e.g., ARID1A, DNMT3A, KMT2 family members), consistent with constitutive signaling and resistance to apoptosis [31,32,33,34,35,36,37]. In parallel, higher tumor cell fraction (TCF) is associated with worse clinical outcomes in MF; TCF thresholds of >25% have been linked to inferior progression-free survival in human cohorts [38].

A defining immunologic transition in advanced MF is the shift from a Th1-associated milieu toward Th2 dominance. Tumor-stage or transformed MF and SS are enriched for Th2 transcriptional programs (e.g., GATA3, STAT3/5/6) with increased IL-4, IL-5, IL-10, IL-13 and reduced IFN-γ and IL-12 signals that weaken cytotoxic immunity over time [14,15,16,18]. Mechanistically, IL-4 and IL-13 can activate STAT6 signaling and promote fibroblast-derived periostin, which, in turn, supports malignant T-cell proliferation and tissue remodeling [14,15]. Guenova et al. provided functional evidence for malignant-clone-driven Th2 enforcement: Th1 responses could be restored when benign T cells were separated from malignant clones or when IL-4/IL-13 were neutralized with specific antibodies [15]. In parallel, rising IL-10 in advanced MF/SS suppresses Th1 responses while enhancing Th2 differentiation, and neutralizing IL-10/IL-10R has been proposed to suppress tumor growth in experimental settings [39]. Transcriptional and epigenetic regulators further reinforce this polarization: in malignant T cells, reduced SATB1 de-represses Th2-associated cytokine genes (e.g., IL-5 and IL-9) at the expense of Th1 programs, and JAK/STAT-linked microRNA circuitry (notably miR-155, reported mainly in MF [40]) has been connected to advanced disease biology and poorer outcomes in human studies [19].

Cytotoxic surveillance fails in tumor-stage MF due to numeric and functional loss of effector cells and increased inhibitory signaling. There is a stage-dependent decline in CD8+ T cells and evidence of exhaustion programs, including increased expression of checkpoint ligands (e.g., PD-L1) within the tumor microenvironment [11,41].

Chemokine trafficking and microenvironmental remodeling become increasingly prominent as disease advances. CCR4 is expressed on the malignant clone across MF and SS rather than being restricted to tumor stage, but the frequency of CCR4-positive cells and the intensity of the CCL17/CCL22 (TARC/MDC) ligand milieu increase with tumor burden, reinforcing skin recruitment and compartmental retention [6,42,43].

Large-cell transformation (LCT) represents an aggressive progression of MF (and less commonly SS), defined by atypical large CD30+ T cells (≥4× small lymphocytes) and occurring in 10–25% of MF cases, with rapid growth and worse prognosis. Clinically, favorable prognostic correlates include normal LDH levels and CD30 expression in >10% of tumor cells, whereas adverse factors include age >60 years, elevated LDH, loss of CD7/CD26, prior SS, and advanced stage at the time of transformation [7,44]. Biologically, LCT demonstrates distinct features, including reduced expression of skin-homing markers (CLA, CCR4), persistent loss of pan-T-cell antigens (e.g., CD2, CD5, CD7) [45], enrichment of regulatory T cells and inflammatory macrophages, and more pronounced activation of JAK/STAT and NF-κB pathways [31,44,45,46,47,48,49,50,51,52]. Notably, enrichment of TNFR2-related signaling has been observed in LCT, correlating with higher mutational burden and poorer overall survival [51]. LCT also shows increased frequency of RAS mutations and broad epigenetic dysregulation, including alterations in TET2, DNMT3A, KMT2D, EP300, EZH2, and CREBBP, compared with non-transformed MF [52].

## 3. Blood Compartment

### SS: Systemic Th2 Dominance, Clonal Dominance, and Cytotoxic Collapse

SS is defined by malignant T-cell circulation and systemic immune derangement. In contrast to TRM-like programs described in MF, SS malignant cells are often characterized as central memory T cells (TCM) expressing CCR4, CCR7, and L-selectin, supporting recirculation between blood, skin, and lymphoid tissues and providing a biologic rationale for distinct clinical behavior across MF vs. SS [20,21,22,53]. High-throughput TCR sequencing in blood allows quantification of dominant circulating clones and longitudinal tracking of clonal evolution, offering a compartment-appropriate measure of clonal dominance over time [54,55,56,57,58].

Genomic analyses (WES/WGS) of SS show frequent TP53 mutations, dysregulated JAK/STAT and NF-κB signaling, and recurrent chromatin remodeling defects, consistent with genomic instability and altered immune signaling within the malignant clone [33,34,35,36,37,48,59]. These lesions map onto both malignant-intrinsic vulnerabilities (therapeutic targets) and malignant-driven immune remodeling.

Systemically, SS exhibits intense Th2 polarization with elevated IL-4/IL-5/IL-13/IL-10 and depressed Th1-associated cytokines such as IFN-γ [12,13,14,15,16,18,19]. Importantly, the human-sample Treg evidence in MF/SS is conflicting itself: reports variably describe higher FOXP3+ densities in early lesions (favorable), no consistent stage-dependent shift, or a paucity in SS, and because neoplastic cells can express FOXP3 themselves and gain or lose it during progression, FOXP3+ counts may conflate reactive and malignant cells [60,61,62]. The prognostic significance of Tregs in MF and SS, therefore, remains unsettled and should be interpreted cautiously.

In SS, circulating NK cells are often reduced relative to healthy donors; the relationship between number of NK cells and survival is unsettled; however, the available data are mixed and at least one predominantly MF cohort linked higher rather than lower NK-cell counts to worse outcomes [10,63,64].

In tumor-stage or transformed MF, accumulating genomic lesions converge on core oncogenic pathways (TCR signaling, JAK/STAT, NF-κB, chromatin modifiers) that promote survival and immune escape, coinciding with emergence of a Th2-dominant state characterized by IL-4/IL-13–STAT6 signaling, stromal remodeling (e.g., periostin), and increased IL-10 in the skin with parallel erosion of Th1 signals [11,14,15,16,17,18,19,31,32,33,34,35,36]. In SS, a distinct blood-centered state, systemic Th2 polarization and cytotoxic failure are evident in blood and skin, with dominant circulating clones and high checkpoint activity [11,12,13,14,15,16,17,18].

Compartmental discordance is clinically relevant: therapies may produce deep blood responses with limited skin benefit (or vice versa) depending on antigen accessibility, stromal remodeling, and the availability of effector-cell substrate. This discordance, however, is inferred largely from selected clinical observations rather than systematic compartment-matched studies. Therefore, biomarker strategies are most informative when compartment-aware, pairing (a) quantitative clonal metrics (e.g., TCF in skin and dominant clone tracking in blood by high-throughput TCR sequencing) with (b) immune competence readouts that directly reflect cytotoxic capacity in the same compartment (e.g., CD8 density/interferon-associated signatures in skin; NK counts and circulating cytokine bias in blood) [38,54,55,56,57,58,65].

The compartment- and stage-associated immune programs discussed above are summarized in Table 1. This framework is not intended as a universal or strictly linear trajectory. Immune remodeling in MF and SS varies with disease stage, compartment, molecular subtype, prior therapy, tumor burden, and the assay platform used for measurement, and not all patients progress through every described state. Many of the associations described here derive from cross-sectional analyses or small cohorts and require prospective, compartment-matched validation before they can be regarded as established.

**Table 1:** Compartment-specific immune programs across CTCL stages (skin and blood).

Compartment/Stage	Assay Types (Examples)	Key Immune Findings (Summary)	Interpretation (Candidate Mechanistic Frame)	Sampling Structure	Strength of Human-Sample Evidence	Refs
Skin: patch/plaque MF	IHC, flow, cytokines; RNA-seq/scRNA-seq (when available)	TRM-like malignant programs; Th1-leaning signals (IFN-γ/IL-12); higher CD8 TIL density; regulatory circuits variably preserved	Locally constrained inflammation with partial cytotoxic containment	Predominantly cross-sectional, skin-only cohorts; generally unpaired with blood; limited longitudinal sampling	Strong (Th1/CD8-TIL-associated observation reproduced across independent early-MF cohorts)	[12,20,21,22,24,25,53]
Skin: patch/plaque MF	TCR clonality (PCR/BIOMED-2; NGS)	Dominant clone may be of low frequency/oligoclonal; NGS improves sensitivity vs. PCR	Early diagnosis benefits from integrated molecular and clinicopathologic assessment	Cross-sectional diagnostic cohorts, usually skin-only and unpaired	Moderate–strong (diagnostic-clonality findings replicated; biological interpretation of low frequency/oligoclonality is less uniform)	[54,55,56,57,58]
Skin: tumor-stage MF	Cytokines; multiplex IHC	Shift to Th2 cytokines (IL-4/IL-5/IL-13/IL-10) with reduced Th1 signals	Polarization supports immune evasion and tumor-promoting inflammation	Predominantly cross-sectional, stage-stratified skin cohorts; usually unpaired with blood; functional ex vivo studies in selected cohorts	Strong (Th2-associated shift reproduced across multiple human cohorts with functional support)	[12,13,14,15,16,17,18,19,39]
Skin: tumor-stage MF	Genomics (WES/WGS/targeted), RNA-seq	Recurrent lesions in TCR signaling, JAK/STAT, NF-κB, chromatin modifiers; higher TCF associates with worse outcomes	Malignant-intrinsic signaling amplifies survival and drives microenvironment remodeling	Cross-sectional tumor-biopsy cohorts; mostly unpaired; limited longitudinal or matched progression samples	Moderate–strong (recurrent pathway lesions replicated; TCF threshold supported by fewer cohorts)	[31,32,33,34,35,36,37,38]
Skin: tumor-stage MF/LCT	IHC; genomics; transcriptomics	CD30 upregulation; TNFR2 signaling enrichment; higher mutation burden; epigenetic dysregulation	Aggressive clonal evolution with immune suppression and therapeutic targetability (CD30)	Retrospective cross-sectional transformed-tumor cohorts; occasional comparison with non-transformed MF; generally unpaired with blood	Moderate (smaller LCT cohorts; TNFR2 enrichment from limited data requiring replication)	[7,31,43,44,45,46,48,49,50,51,52]
Skin and blood: advanced MF/SS	Checkpoint IHC/RNA; immune profiling	PD-1/PD-L1 axis upregulation; exhaustion programs	Inhibitory signaling contributes to cytotoxic failure; candidate target for ICIs	Cross-sectional skin and/or blood cohorts, frequently mixed MF/SS; paired skin–blood analyses uncommon	Moderate (several cohorts; but mixed diagnoses, semiquantitative assays, and limited compartment matching)	[11,41,66,67,68,69]
Skin and blood: CTCL (variable by cohort)	NK phenotyping; functional assays	NK numeric/functional abnormalities; inhibitory-receptor changes; assay-dependent discrepancies; NK counts associated with OS (direction unsettled across cohorts)	Cytotoxic compartment failure; methodological factors matter	Cross-sectional skin or blood cohorts with heterogeneous MF/SS composition; generally unpaired; assay-dependent results	Limited (discordant findings across heterogeneous cohorts and assays)	[10,63,70,71,72,73,74]
Blood: SS	Flow cytometry; TCR PCR; qRT-PCR; miRNA profiling; WES/RNA-seq	Circulating malignant clone; Th2-associated transcriptional skew; distinct miRNA profiles; recurrent T-cell signaling and cell-cycle alterations	Quantifiable leukemic compartment with diagnostic and targetable molecular abnormalities	Predominantly cross-sectional, blood-only cohorts; validation cohorts available; limited longitudinal or paired skin–blood sampling	Moderate–strong (consistent blood-based molecular findings; individual signatures require further validation)	[40,47,55,65]

Strength of human-sample evidence is graded according to replication, consistency, cohort size and independence, compartment/stage resolution, and functional corroboration, as defined in Section 1.1. The grade applies to the biological observation and not merely to the maturity of the assay. Sampling structure is reported separately to show whether evidence is cross-sectional or longitudinal and paired or unpaired across skin and blood. Abbreviations: BIOMED-2, European BIOMED-2 TCR/Ig clonality primer sets; CD, cluster of differentiation; CTCL, cutaneous T-cell lymphoma; ICI(s), immune checkpoint inhibitor(s); IFN-γ, interferon-gamma; IHC, immunohistochemistry; IL, interleukin; JAK/STAT, Janus kinase/signal transducer and activator of transcription; LCT, large-cell transformation; MF, mycosis fungoides; NF-κB, nuclear factor-kappa B; NGS, next-generation sequencing; NK, natural killer; OS, overall survival; PCR, polymerase chain reaction; PD-1, programmed cell death protein 1; PD-L1, programmed death-ligand 1; RNA-seq, RNA sequencing; scRNA-seq, single-cell RNA sequencing; SS, Sézary syndrome; TCF, tumor cell fraction; TCM, central memory T cells; TCR, T-cell receptor; Th1/Th2, T helper 1/2; TIL(s), tumor-infiltrating lymphocyte(s); TRM, tissue-resident memory T cells; WES/WGS, whole-exome/whole-genome sequencing.

## 4. Therapeutic Implications of Compartment-Aware Biology in MF and SS

We group the targets below by the maturity of their supporting evidence rather than by molecular family. Established, regulatory-approved options supported by mature prospective data include mogamulizumab, brentuximab vedotin, and denileukin diftitox-cxdl. Agents with phase II or robust early-phase efficacy include lacutamab and PD-1 blockade (e.g., pembrolizumab), alongside extracorporeal photopheresis, which is widely used for erythrodermic and leukemic disease but supported largely by observational data. Earlier-stage or investigational strategies include CD47, CD70, TNFR2, and JAK/STAT-directed approaches, together with cellular therapies (CD5-, CD70-, and CD30-directed CAR-T). For each target we indicate the compartment in which activity is most expected and the candidate biomarkers that might guide selection; Table 2 summarizes these agents together with the strength of supporting evidence and current regulatory status.

### 4.1. CCR4 Targeting (Mogamulizumab): Clone Depletion and Treg Modulation

CCR4 is a skin-homing receptor expressed on malignant CD4+ T cells and on subsets of regulatory T cells (Tregs), supporting migration along CCL17/CCL22 gradients. Mogamulizumab is a defucosylated anti-CCR4 monoclonal antibody that enhances FcγRIIIa-mediated antibody-dependent cellular cytotoxicity (ADCC), producing (i) direct depletion of CCR4+ malignant clones and (ii) partial depletion of CCR4+ Tregs. Clinically, in the phase III MAVORIC trial comparing mogamulizumab vs. vorinostat in previously treated MF/SS (n = 372), mogamulizumab achieved a superior median PFS (7.7 months vs. 3.1 months), consistent with robust activity in relapsed MF/SS where circulating malignant cells are readily accessible for ADCC-mediated clearance. In MAVORIC, response segregated sharply by compartment—blood, 68%; skin, 42%; and lymph node, 17%—with a higher global response in SS than MF (37% vs. 21%) and exclusion of large-cell transformation. Real-world cohorts reproduce this blood-over-skin hierarchy (skin ~43% vs. blood ~90%) and additionally link mogamulizumab to improved overall survival in SS that the crossover-confounded trial could not be established (adjusted hazard ratio 0.34 in a 339-patient European cohort) [75,76,77]. Because the malignant clone is almost universally CCR4-positive, CCR4 expression level did not distinguish responders from non-responders in either the trial or real-world practice [78], so CCR4 density should not gate therapy, and skin responses concentrate in blood-involved and erythrodermic disease rather than early skin-limited MF. Potential, as-yet-unvalidated biomarkers to explore include CCR4 mutations and baseline ADCC effector capacity (Table 2).

### 4.2. CD30 Targeting (Brentuximab Vedotin): Antigen-Dependent Cytoreduction with Secondary Immune Effects

CD30 is commonly upregulated in tumor-stage MF and in LCT. Brentuximab vedotin (BV) is an anti-CD30 antibody–drug conjugate that delivers monomethyl auristatin E (MMAE) to CD30-expressing cells, leading to antigen-dependent cytoreduction [66,79]. In the phase III ALCANZA trial (BV vs. physician’s choice), BV significantly improved objective response lasting ≥4 months (55% vs. 13%), complete response (17% vs. 2%), and PFS (16.7 vs. 3.5 months), establishing CD30-directed cytoreduction as a key systemic option in CD30-expressing CTCL [66].

Because BV primarily reduces malignant burden rather than directly reprogramming Th1 immunity, durability depends on whether cytotoxic surveillance can reassert control once tumor-derived Th2 cytokines and suppressive signals decline. ALCANZA enrolled CD30-positive MF (≥10% CD30 on ≥1 biopsy) and primary cutaneous ALCL but excluded SS; within it, response lasting ≥4 months was independent of CD30 level (41% at CD30 <10% vs. 57% at ≥10%) and was at least as high with large-cell transformation as without (65% vs. 39%), positioning brentuximab vedotin as a preferred option for transformed and tumor-stage disease [80]. Real-world data extend activity below the eligibility threshold (overall response of 53% in advanced MF/SS with CD30 <10%), reinforcing that CD30 immunohistochemical density predicts response poorly and should not exclude patients [81,82]. Potential, as-yet-unvalidated biomarkers to explore are antigen-centric, chiefly CD30 expression and its lesional heterogeneity between lesions, which is most relevant in tumor-stage MF and large-cell transformation (Table 2).

### 4.3. IL-2 Receptor Targeting (CD25 Axis): Denileukin Diftitox-Cxdl (Reformulated) and Related Agents

CTCL malignant clones and tumor-associated regulatory T-cell programs can express IL-2 receptor α (CD25), creating an opportunity for selective depletion using IL-2-based fusion toxins. Denileukin diftitox-cxdl is a reformulated IL-2–diphtheria toxin fusion protein designed to improve manufacturing consistency and reduce immunogenic contaminants relative to earlier products.

In the pivotal randomized phase III multicenter, open-label, single-arm Study 302 in relapsed/refractory CTCL, denileukin diftitox-cxdl achieved an overall response rate of 36.2% (including complete responses) with a median duration of response of 8.9 months [83]. Eligibility required CD25 expression on ≥20% of malignant cells, and the treated population was almost entirely MF spanning stages I–III; unlike CCR4 and CD30, CD25 density does appear to track with response (historically ~79% in CD25-high vs. ~20% in CD25-low disease), yet meaningful responses still occur below the threshold (overall response of ~31% in CD25-low CTCL), so even here expression predicts but does not dictate benefit [84,85]. On the basis of these data, denileukin diftitox-cxdl received U.S. FDA approval in August 2024 for relapsed/refractory CTCL after at least one prior systemic therapy and has subsequently been incorporated into NCCN guidelines (category 2A). Similarly, Kawai et al. demonstrated clinical efficacy of E7777 (denileukin diftitox) in relapsed/refractory CTCL with an ORR of 31% and median PFS of 4.2 months in a phase II study [86]. These data support IL-2R-targeted depletion as a viable axis in advanced CTCL where both malignant clones and immunosuppressive CD25+ subsets may contribute to disease persistence.

Mechanistically, IL-2R targeting sits at the intersection of clone depletion and immunosuppression relief: it can directly kill CD25+ malignant cells and reduce CD25+ regulatory compartments, which may enable endogenous cytotoxic recovery when an effector substrate is still present. Potential, as-yet-unvalidated biomarkers to explore include CD25 expression and baseline immune competence, across skin and blood disease (Table 2).

### 4.4. Cytokine Reprogramming (IFN and IL-12): Restoring Th1 Polarization and Cytotoxic Recruitment

Multiple human studies support a stage-dependent decline in IFN-γ-associated programs as MF advances, with progressive Th2 polarization in tumor-stage or transformed MF and SS characterized by increased IL-4/IL-13/IL-5 signaling and STAT-mediated transcriptional reinforcement [9,10,14,15,16,87]. IFN-γ and IL-12 strategies can be conceptualized as “cytokine reprogramming” interventions that aim to restore Th1 polarization and strengthen cytotoxic recruitment and function. Mechanistically, IL-12 can promote Th1 differentiation and activate NK and CD8+ response. IFN-γ also integrates into trafficking loops (e.g., IFN-γ-induced keratinocyte IP-10/CXCL10), which may influence epidermotropism and immune cell recruitment in CTCL lesions, while IFN-γ-associated signals decline with progression [12,13].

Clinically, the interferon with substantial activity is IFN-α rather than IFN-γ, producing historical skin responses of roughly 40–80% in MF, highest in early-stage and lower in tumor-stage disease, without a predictive tissue biomarker; because recombinant IFN-α is no longer manufactured, contemporary practice relies on pegylated IFN-α-2a, with an overall response near 53% across early and advanced stages and long time-to-next-treatment in combination [88]. Recombinant IL-12 was evaluated only in small early-MF series (lesion regression with restored CD8/NK activity) and was not developed further. Potential, as-yet-unvalidated biomarkers to explore include preserved interferon-pathway competence and a measurable baseline cytotoxic infiltrate, most relevant in early patch/plaque skin disease (Table 2).

### 4.5. Extracorporeal Photopheresis (ECP): Immune Modulation in Erythrodermic/Blood-Dominant CTCL

ECP is a standard immune-modulatory intervention in erythrodermic CTCL, particularly SS [89,90]. ECP induces apoptosis to a portion of circulating leukocytes followed by reinfusion; the prevailing model is that uptake of apoptotic material and altered antigen presentation promotes immunologic ‘re-education’ rather than direct cytotoxic elimination. Accordingly, ECP is biologically aligned with erythrodermic or blood-involved disease where malignant clones are measurable and serial immunomonitoring is feasible [38,55,56,57,58,91].

Consistent with this mechanism, extracorporeal photopheresis is effective in erythrodermic and leukemic disease (pooled response of ~63% in erythrodermic CTCL) but has little role in tumor-stage MF, and response is predicted by lower blood tumor burden and circulating Sézary count rather than by any antigen [89,90]. Skin and blood responses track together but evolve slowly (often over ~6 months), and registry data associate ECP—like mogamulizumab and interferon—with longer survival and time-to-next-treatment in SS [75]. Potential, as-yet-unvalidated biomarkers to explore include circulating clonal burden by TCR sequencing and preserved CD8/NK compartments in erythrodermic/blood-dominant (Sézary) disease (Table 2).

### 4.6. PD-1/PD-L1 Axis Inhibition: Reinvigoration of Exhausted Effectors with Malignancy-Specific Complexity

Immune checkpoint blockade has biologic rationale in CTCL because advanced MF/SS show exhaustion programs and PD-1/PD-L1 pathway upregulation, alongside progressive loss of functional cytotoxic substrate [11]. However, CTCL is a T-cell malignancy: PD-1 is expressed on benign effectors and can also be expressed on malignant T cells, creating the possibility of mixed biologic effects. Clinically, pembrolizumab demonstrated activity in relapsed/refractory MF/SS (ORR of 38% in CITN-10), supporting the model that a subset of patients retains reversibly exhausted CD8+ T cells and/or NK cells that can be re-engaged [67]. Early combination data with PD-L1 blockade plus immunomodulation (durvalumab + lenalidomide) also showed meaningful clinical activity (reported ORR of 58.3% in relapsed/refractory CTCL), reinforcing the concept that “immune substrate + activation context” may be more informative than PD-1 expression alone [68].

In CITN-10, responses did not correlate with PD-L1 expression, tumor mutational burden, or an interferon-γ signature, and 23 of 24 patients had advanced (stages IIB–IV) disease; clinically notable is a transient skin/erythroderma flare in roughly half of Sézary patients that tracks with high PD-1 on the malignant clone rather than with response [67]. Because PD-1 can be expressed by the malignant T cell itself, checkpoint blockade carries a real-world risk of paradoxical progression in blood-involved disease, so PD-L1 density is neither a reliable response biomarker nor a safe selection gate. Potential, as-yet-unvalidated biomarkers to explore include lesional CD8/NK abundance and interferon-stimulated gene signatures, rather than PD-1 expression alone, with relevance to advanced skin disease (Table 2).

### 4.7. KIR3DL2 Targeting: Lacutamab

KIR3DL2 (CD158k) is a surface receptor enriched on Sézary cells and present in a substantial subset of MF, with limited expression in most non-malignant tissues. Lacutamab is an anti-KIR3DL2 monoclonal antibody engineered to drive antibody-dependent cellular cytotoxicity (ADCC), using Fc-receptor-bearing effector cells (especially NK cells) to deplete KIR3DL2+ malignant clones. In the phase II TELLOMAK trial, lacutamab demonstrated activity in relapsed/refractory MF and SS, with cohort-specific outcomes. In the SS cohort, the global objective response rate (ORR) was 37.5% (skin, 46.4%; blood, 48.2%), and longer-term follow-up reported a global ORR of 42.9% with a median duration of response of 25.6 months. In the MF cohort, the global ORR was 19.6% (Olsen 2011 criteria) with a skin response of 29.0%; responses were observed across KIR3DL2 expression levels (ORR of 20.8% in patients with KIR3DL2 ≥1%), supporting target expression as a candidate enrichment variable rather than a strict requirement [92,93,94,95,96].

Mechanistically, lacutamab sits at the intersection of clone depletion and effector engagement. Large-cell transformation was excluded from TELLOMAK, and, uniquely among these agents, KIR3DL2 is used prospectively for cohort allocation, making lacutamab the cleanest ongoing test of whether antigen density tracks with response. Potential, as-yet-unvalidated biomarkers to explore include KIR3DL2 target density and an ADCC effector substrate, with deeper responses expected in blood/Sézary than in skin (Table 2).

**Table 2:** Compartment-aware immunotherapies and targeted immune strategies in CTCL.

Therapy (Target)	Compartment/Stage	Primary Immune Mechanism	Strongest Clinical Evidence (Design, N, Key Result)	Evidence Tier	Clinical/Regulatory Status	Candidate Predictive Biomarker(s)	Ref(s)
Mogamulizumab (anti-CCR4)	Blood/leukemic (Sézary); also skin	Defucosylated mAb; enhanced ADCC against CCR4+ malignant T cells; partial Treg depletion	Phase III RCT (MAVORIC, n = 372) vs. vorinostat: PFS 7.7 vs. 3.1 mo; global ORR 28%	Established	FDA/EMA-approved (r/r MF/SS); NCCN-recommended	Blood tumor burden (greatest activity in blood); CCR4 mutations; and baseline ADCC effector capacity	[43]
Brentuximab vedotin (anti-CD30 ADC)	Tumor-stage MF; LCT (skin)	Anti-CD30 ADC delivering MMAE; antigen-dependent cytoreduction	Phase III RCT (ALCANZA, n = 128): ORR4 ≈55% vs. 13%; CR 17% vs. 2%; PFS 16.7 vs. 3.5 mo	Established	FDA/EMA-approved (CD30+ MF/pcALCL after prior therapy); NCCN	CD30 expression (activity across levels if ≥10% in ≥1 biopsy); LCT	[66,79]
Denileukin diftitox-cxdl (anti-CD25/IL-2R fusion toxin)	Skin and blood (systemic)	IL-2–diphtheria toxin fusion; depletes CD25/IL-2R+ malignant cells and Tregs	Phase III single-arm (Study 302, n = 69): ORR 36.2% (CR 8.7%); DOR 8.9 mo	Established	FDA-approved (Aug 2024, r/r CTCL after ≥1 systemic therapy); NCCN cat 2A	CD25 expression; soluble IL-2Rα (sCD25)	[83]
Pembrolizumab (anti-PD-1)	Advanced (skin and blood)	PD-1 blockade reinvigorates exhausted CD8/NK effectors	Phase II single-arm (CITN-10, n = 24): ORR 38% (2 CR); durable responses	Emerging	Not CTCL-approved; selected use; caution re: possible rapid progression	Effector substrate (CD8/NK); IFN-inflamed signature; PD-L1/L2 variants	[67]
Lacutamab (anti-KIR3DL2)	Blood/Sézary > skin	ADCC-mediated depletion of KIR3DL2+ malignant cells	Phase II (TELLOMAK): SS ORR 37.5%→42.9% (DOR 25.6 mo); MF ORR 19.6% (skin 29%)	Emerging	Investigational; FDA Breakthrough Therapy (2025, r/r SS); phase III planned	KIR3DL2 expression (flow in blood; IHC in skin)	[92,94,95,96]
Extracorporeal photopheresis (ECP)	Erythrodermic/blood (Sézary)	Immunomodulation of circulating compartment; tolerogenic/apoptotic effects	Observational/retrospective cohorts; long-standing use in erythrodermic SS	Standard use; observational evidence	Established clinical option (NCCN-listed) for erythrodermic/leukemic disease	Blood clonal burden (TCR sequencing); retained immune competence	[75,89,90]
Durvalumab + lenalidomide (anti-PD-L1 + IMiD)	Advanced (skin and blood)	PD-L1 blockade plus immunomodulation (T/NK activation)	Phase I (small): ORR 58%, DOR 25.5 mo; randomized phase II reported	Emerging	Investigational	Composite immune-activation/IFN-inflamed signatures	[68]
IFN-γ	Early skin (patch/plaque)	Th1 reinforcement; CXCL9/10-driven effector recruitment	Small studies/case series; variable responses	Early-investigational	Historical/adjunct use; limited prospective CTCL data	STAT1/IRF1 IFN competence; baseline CD8/NK	[12,25]
IL-12	Early skin (patch/plaque)	Th1 polarization; CD8/NK activation	Early-phase trial: ~50% response (small cohort) with increased CD8/TIA-1 infiltrates	Early-investigational	Investigational (development limited by toxicity)	Baseline cytotoxic infiltrate; IFN/STAT4 signals	[24,97,98]
JAK/STAT inhibitors (e.g., ruxolitinib)	Tumor stage; skin and blood	Block malignant JAK/STAT signaling	Limited CTCL-specific and basket/early-phase data	Early-investigational	Investigational in CTCL	pSTAT3/5; JAK/STAT genomic lesions	[33,99,100]
TTI-621 (CD47/SIRPα blockade)	Skin (lesional)	Innate checkpoint blockade promoting macrophage phagocytosis	Phase I: systemic ORR ~21%; intralesional tumor reduction in ~90%	Early-investigational	Investigational	Tumor CD47; macrophage-rich TME	[101]
Cusatuzumab (anti-CD70)	Advanced (blood and skin)	Anti-CD70 mAb; ADCC/phagocytosis	Phase I/II (n = 27): ORR 23% (incl post-mogamulizumab)	Early-investigational	Investigational	CD70 expression level/uniformity	[102]
BI-1808 (anti-TNFR2)	Skin and blood (Treg-rich)	Depletes/modulates TNFR2+ tumor-associated Tregs	Phase I/IIa: early signals in CTCL cohort	Early-investigational	Investigational; FDA Fast Track + Orphan Drug (2025, r/r MF/SS)	TNFR2 expression; Treg-rich TME (exploratory)	[103,104,105]
E7777 (denileukin diftitox class)	Skin and blood (systemic)	IL-2R-directed fusion toxin (improved purity)	Phase II (Japan, n = 36): CTCL ORR 31% (overall 36%)	Early-investigational	Approved in Japan (Remitoro); precursor to DD-cxdl	CD25 expression	[86]
CD5 CAR-T (autologous)	Advanced (blood and skin)	Adoptive cytotoxicity against CD5+ malignant T cells	Phase I in T-cell malignancies; early responses	Early-investigational (cellular)	Investigational	CD5 retention (often reduced in advanced MF)	[106]
CD70-directed CAR-T (e.g., CTX130, allogeneic)	Advanced (blood and skin)	Adoptive cytotoxicity against CD70+ targets	Phase I (e.g., COBALT-LYM): early signals in T-cell lymphoma	Early-investigational (cellular)	Investigational	CD70 antigen density	[107,108,109]
CD30-directed (CCR4 co-expressing) CAR-T	Tumor stage/CD30+ (skin)	Engineered adoptive cytotoxicity against CD30+ with skin-homing	Preclinical/early development	Preclinical–early	Investigational	CD30 expression; skin-homing chemokine axis	[109,110]
Tenalisib (PI3Kδ/γ inhibitor)	Advanced (skin and blood)	PI3K inhibition; immunomodulatory effects	Phase I/II in T-cell lymphomas incl CTCL; combination data	Early-investigational	Investigational	PI3K pathway activation; cytokine milieu	[93,111]

Evidence tiers: established = regulatory-approved and/or supported by phase III or mature prospective data; emerging = phase II or robust early-phase efficacy; early-investigational = phase I, small, or single-arm signals; preclinical–early = preclinical or first-in-human. The evidence tiers reflect therapeutic development maturity and are distinct from the strength-of-evidence grading applied to biological associations in Table 1. ADCC, antibody-dependent cellular cytotoxicity; CAR-T, chimeric antigen receptor T cells; DD-cxdl, denileukin diftitox-cxdl; DOR, duration of response; LCT, large-cell transformation; MMAE, monomethyl auristatin E; ORR(4), objective response rate (lasting ≥4 months); PFS, progression-free survival; RCT, randomized controlled trial. **Abbreviations:** ADC, antibody–drug conjugate; ADCC, antibody-dependent cellular cytotoxicity; AE(s), adverse event(s); CAR-T, chimeric antigen receptor T-cell therapy; CCR4, C-C chemokine receptor 4; CD, cluster of differentiation; CRS, cytokine release syndrome; CTCL, cutaneous T-cell lymphoma; DC, dendritic cell; ECP, extracorporeal photopheresis; FcγR, Fc gamma receptor; FCGR3A, Fc gamma receptor IIIa (CD16a) gene; IFN, interferon; ICANS, immune effector cell-associated neurotoxicity syndrome; ICI(s), immune checkpoint inhibitor(s); IFN-γ, interferon-gamma; IHC, immunohistochemistry; IL, interleukin; IL-2R, interleukin-2 receptor; IRF1, interferon regulatory factor 1; ISG, interferon-stimulated gene(s); JAK/STAT, Janus kinase/signal transducer and activator of transcription; KIR3DL2, killer cell immunoglobulin-like receptor 3DL2; LCT, large-cell transformation; MF, mycosis fungoides; MMAE, monomethyl auristatin E; NGS, next-generation sequencing; NK, natural killer; OS, overall survival; PD-1, programmed cell death protein 1; PD-L1, programmed death-ligand 1; PI3K, phosphoinositide 3-kinase; pSTAT, phosphorylated STAT; RNA, ribonucleic acid; SIRPα, signal regulatory protein alpha; SS, Sézary syndrome; STAT, signal transducer and activator of transcription; TCF, tumor cell fraction; Th1/Th2, T helper 1/2; TIA-1, T-cell intracellular antigen-1; TME, tumor microenvironment; Treg(s), regulatory T cell(s).

## 5. Discussion

### 5.1. What Is Reasonably Established

Across the skin and blood compartments, MF and SS show a recurring association between disease progression and three coupled changes: expansion of the malignant clone, a relative shift from Th1- toward Th2-associated cytokine programs, and attenuated cytotoxic surveillance. This compartment- and stage-aware description is useful for organizing immune-directed therapy, but it is an interpretive framework rather than a proven causal sequence.

One of the key challenges in MF/SS is the loss of immune control, particularly the shift from a Th1 to a Th2-dominant TME. This transition not only weakens anti-tumor immunity but also fosters an environment where malignant T cells evade immune surveillance. Another hallmark of immune dysregulation in MF/SS is the emergence of regulatory immune cells that support tumor persistence by inhibiting CD8+ T-cell function and suppressing inflammatory responses that might otherwise limit tumor growth. Similarly, Bregs, through IL-10 production, further dampen anti-tumor immunity, promoting a permissive environment for malignant T cells to thrive [70,112]. This has motivated interest in approaches that target not only the malignant T cells but also the supportive tumor microenvironment. Strategies to restore Th1 responses, such as IFN therapy, have shown promise but require further refinement in timing and doses to ensure sustained immune activation without excessive toxicity. Similarly, immune checkpoint inhibitors (ICIs), such as pembrolizumab and durvalumab, have demonstrated meaningful clinical responses in some patients with advanced CTCL, though response rates remain variable [67,113].

### 5.2. Limitations and Open Questions

Several limitations temper this framework. First, much of the compartment- and stage-specific immune data are derived from small, cross-sectional, or single-center cohorts using heterogeneous assays, so apparent trajectories may partly reflect sampling and methodology rather than biology; longitudinal, compartment-matched studies remain scarce. Second, malignant cells and target antigens are modulated under selective pressure, and skin–blood discordance in target expression complicates both prediction and monitoring. Third, none of the candidate predictive biomarkers discussed here is validated for treatment selection in MF or SS yet; most rest on retrospective or mechanistic associations and require prospective, ideally biomarker-stratified, evaluation. Fourth, comparative evidence is limited: outside MAVORIC and ALCANZA, few randomized or head-to-head data exist, and several promising agents are supported only by single-arm signals in heavily pretreated patients. Finally, external validity is constrained by the demographics of existing trials; reported enrollment in U.S. interventional MF/SS studies has included relatively few Black participants despite a higher age-adjusted incidence and worse outcomes in this group, and stage, subtype, and prior-therapy mix vary across cohorts. Demographic-stratified reporting and broader, more representative enrollment are needed before biomarker- and compartment-guided strategies can be assumed to generalize.

Advancements in genomic and transcriptomic profiling have substantially advanced the molecular characterization of CTCL, informing the study of tumor heterogeneity, clonal evolution, and resistance mechanisms. High-throughput sequencing studies have identified recurrent single-nucleotide variants (SNVs) in TP53, ZEB1, ARID1A, DNMT3A, CDKN2A, FAS, ATM, CTCF, TNFAIP3, STAT5B, PRKCQ, and IRF4, as well as mutations in pathways involved in chromatin remodeling (ARID1A, DNMT3A, KMT2C), cell cycle regulation (CDKN2A, RB1), apoptosis (FAS, TNFRSF10A), MAPK signaling (KRAS, BRAF, MAPK1), and DNA damage repair (ATM, TP53) [11,35,51,114,115,116]. While these findings provide new molecular targets for therapy, emerging mechanisms of resistance necessitate further refinement of these targeted therapies. The application of NGS, including scRNA-seq, could further differentiate malignant clones from reactive immune populations, ultimately guiding more personalized, adaptive treatment strategies.

### 5.3. Priorities for Future Studies

Several questions remain unresolved. The extent to which the compartment- and stage-associated cytokine trajectories described here are universal rather than cohort-specific is unknown, as is the degree to which skin and blood findings can be extrapolated to one another. Patient-to-patient variation in response to immune-directed therapy is not yet explained by available biomarkers, and the relative contributions of tumor biology, time to diagnosis, comorbidity, and access to observed outcome differences remain difficult to separate. Resolving these questions will depend on larger, prospectively collected, and demographically representative cohorts with standardized, compartment-matched immune and clonal measurements.

In summary, MF and SS are best understood as compartmentalized diseases in which immune dysregulation evolves with stage and site. A compartment- and stage-aware reading of the biology offers a rational basis for matching immune-directed therapies to disease context and for prioritizing candidate biomarkers, but most of those biomarkers are not yet validated and few therapies have been compared directly. The central task for the field is therefore disciplined, prospective, and biomarker-stratified evaluation in representative populations, so that the mechanistic rationale outlined here can be tested rather than assumed.

## 6. Conclusions

MF and SS are compartmentalized T-cell malignancies in which immune dysregulation varies according to disease subtype, stage, and anatomic site. Early patch/plaque MF is generally associated with skin-resident malignant clones, a relatively Th1-permissive microenvironment, and partially preserved cytotoxic surveillance. Tumor-stage and transformed MF demonstrate increasing clonal dominance, Th2 polarization, checkpoint activation, and stromal and myeloid remodeling. SS is distinguished by circulating malignant clones, systemic Th2 skewing, and broader impairment of cytotoxic immune control.

This biological heterogeneity provides a rationale for matching immune-directed therapies to the dominant disease compartment and available effector-cell substrate. Established approaches targeting CCR4, CD30, or CD25, together with ECP and interferon-based therapy, illustrate how therapeutic activity can differ between skin and blood. Emerging strategies targeting KIR3DL2, immune checkpoints, JAK/STAT signaling, and other tumor–microenvironment interactions may further expand treatment options. However, most proposed predictive biomarkers, including antigen density, clonal or tumor burden, cytotoxic-cell abundance, and interferon-related signatures, remain exploratory.

Future progress will require prospective, longitudinal studies incorporating paired skin and blood samples, standardized immune and clonal measurements, and representative patient populations. Such studies should determine whether compartment- and stage-associated biomarkers can reliably guide treatment selection, monitor response, and identify resistance. Until these biomarkers are validated, compartment-aware assessment should be regarded as a biologically informed framework for clinical investigation rather than a definitive treatment-selection algorithm.

## Data Availability

No new data were created or analyzed in this study. Data sharing is not applicable to this article.
